# Birth Cohort Changes in the Subjective Well-Being of Chinese College Students: A Cross-Temporal Meta-Analysis, 2002–2017

**DOI:** 10.3389/fpsyg.2020.01011

**Published:** 2020-06-11

**Authors:** Qian Su, Guofang Liu

**Affiliations:** ^1^Faculty of Psychology, Beijing Normal University, Beijing, China; ^2^School of Economics and Management, Shanghai Maritime University, Shanghai, China

**Keywords:** birth cohort, subjective well-being, cross-temporal meta-analysis, Chinese college students, social change

## Abstract

According to the happiness-income paradox, economic growth within a country does not necessarily lead to an increase in well-being. However, previous literature also showed that economic growth has a greater impact on well-being in a low-income country than a high-income country. China is a typical developing country that has experienced dramatic development in recent decades. How did the well-being of the Chinese change? To examine birth cohort changes in Chinese college students' subjective well-being, a cross-temporal meta-analysis that involved 100 studies was conducted (106 data points, *N* = 55,830). The results showed that Chinese college students' well-being increased by at least 0.45 standard deviations from 2002 to 2017. In addition, their subjective well-being was significantly correlated with social indicators (e.g., GDP per capita, divorce rate, and university enrollment rate) for the corresponding years and 3 years prior to the collection of subjective well-being data. It is evident that social changes play an important role in predicting changes in well-being.

## Introduction

Economic growth and human well-being are two essential goals that both countries and individuals strive for. However, achieving both goals is difficult. For example, China has experienced dramatic economic growth during the past decades, and its GDP is now the second highest in the world. In contrast, the well-being level of the Chinese ranks in the middle of countries throughout the world (Helliwell et al., 2012). Some positive indicators of the mental health of Chinese people, such as social support and self-esteem, have also shown decreases in recent decades (Sha and Zhang, 2016; Xin and Xin, 2016). The evidence prompts the question of how the subjective well-being (SWB) of the Chinese has changed. This study thus investigated birth cohort changes in Chinese college students' SWB from 2002 to 2017.

Investigating the relationship between economic growth and SWB is an active research domain in economics and psychology. Based on cross-sectional data, a series of studies revealed that the rich are happier than the poor (e.g., Tong, 2003; Luo and Li, 2008; Ding and Wang, 2011; Malkoc, 2011; Vahedi and Nazari, 2011; Watson et al., 2015; Castillo-Lavergne and Destin, 2019). For example, Easterlin (1974) revealed that Americans with higher incomes were more likely to report feeling happy or very happy than those with lower incomes. Yan et al. (2002) found that family income was positively correlated with college students' SWB, and participants who came from high-income families reported higher levels of well-being than those from low-income families. Hence, income seems to be important for well-being.

Although the rich are frequently happier than the poor, we cannot expect that increases in income necessarily improve well-being. Easterlin (2003) proposed that the answer to “does happiness go together with income” depends on whether one looks at cross-sectional or time series data. On the one hand, the happiness-income paradox suggests that although the rich will report higher levels of happiness than the poor within a country, over the long term, happiness does not increase as a country's income rises (Easterlin, 1974, 2003; Easterlin et al., 2010). For example, analysis of World Values Survey data showed that the relationship between SWB and the annual rate of GDP growth is nil (Easterlin et al., 2010). Further, over the life cycle, as income increases and then levels off, happiness remains unchanged (Easterlin, 2001, 2003). Accordingly, it seems that increases in income do not affect SWB.

However, in Easterlin and Sawangfa's (2009) study, the life satisfaction of participants in nine of twelve countries exhibited an upward trend. A regression analysis also showed that SWB in three of six countries (including Mexico and Venezuela) was significantly correlated with log GDP. To explain this phenomenon, Easterlin and Sawangfa proposed that “a given absolute increase in GDP per capita has a greater impact on SWB in a low-income than in a high-income country.” As mentioned above, the GDP in China is now the second highest in the world. However, the GDP per capita in China is still relatively low. According to the (World Bank, 2019), China's GDP per capita was ~1,148 dollars in 2002 (compared with ~38,023 dollars for the United States) and 9,770 dollars in 2018 (compared with ~62,641 dollars for the United States). According to the report of the National Bureau of Statistics of China (2018), the poverty rate of rural China had decreased from 49.8% in 2000 to 3.1% in 2017. Therefore, China is a developing country that has made great progress. According to Easterlin and Sawangfa's (2009) view, it is reasonable to hypothesize that China's economic growth during the past decades would improve the SWB of Chinese people.

Indeed, some evidence suggests that the Chinese have experienced an increase in well-being. Dong (2016) conducted a cross-temporal meta-analysis on the SWB of China's urban residents and revealed that the SWB of China's urban residents increased from 2003 to 2014. In the Inglehart et al., (Inglehart et al., 2014), SWB was measured by two items: “Taking all things together, would you say you are very happy, quite happy, not very happy, or not at all happy?” and “All things considered, how satisfied are you with your life as a whole these days?” The former and latter items represent affective and cognitive aspects of SWB, respectively (Easterlin and Sawangfa, 2009; Minkov, 2009; Bartolini et al., 2017). For the former item, the percentages of participants who reported being very happy or quite happy in 1990, 1995, 2001, 2007, and 2013 were 66.6, 83.6, 77.8, 76.8, and 84.5, respectively. The second item was measured on a Likert scale from 1 to 10, with higher scores indicating higher life satisfaction. The mean scores (and standard deviations, *SDs*) of Chinese participants in the five waves were 7.29 (2.10), 6.83 (2.42), 6.53 (2.47), 6.76 (2.43), and 6.85 (1.98). The World Values Survey data also showed that the SWB of Chinese people increased from 2001 to 2013.

Despite the mentioned evidence of changes in Chinese SWB (Inglehart et al., 2014; Dong, 2016), it needs to be investigated further. On the one hand, China has only five data points in the World Values Survey, which may misrepresent the true chronological trend in SWB. SWB was affected by major events that participants experienced recently (Bernat et al., 1998; Gabert-Quillen et al., 2015). For example, hosting the Olympic Games increased residents' SWB (Dolan et al., 2019), and huge natural disasters such as earthquakes decreased residents' SWB (He, 2013). During the past decades, China has experienced both massive positive and negative social events such as the Wenchuan earthquake and Beijing Olympics Games in 2008. Therefore, it is difficult to reflect the long-term change in SWB with the evidence from a few years. On the other hand, although Dong (2016) analyzed year-by-year data regarding the SWB of Chinese urban residents, it is unknown whether the increasing trend for a Chinese sample that Dong revealed exists in other Chinese samples. Therefore, the current study aimed to investigate the chronological trends in the SWB of Chinese college students from 2002 to 2017.

There are three reasons to investigate birth cohort changes in college students' SWB. First, previous research found that SWB is influenced by the comparative reference that a person chooses (Bai et al., 2009; Zhuo, 2010; Chou and Edge, 2012; Zhang, 2012; Haught et al., 2015). In contrast to general civilizations, the social status and incomes of college students are relatively balanced. Therefore, birth cohort changes in college students' SWB should be investigated further. Second, a nationwide survey of China in 2018 showed that participants who were born in the 1990s had the lowest SWB (Wang and Chen, 2018). Although we cannot identify the specific professions of these individuals, many are college students. Therefore, the results may indicate that college students have lower SWB. Finally, in our view, college students are the group that is most likely to influence social development in the future. Therefore, recently in China, many big cities such as Beijing, Shanghai, and Hangzhou began to compete for college students (CCTV news, 2019a). SWB has also been found to be an important factor for career success and productivity (Staw and Barsade, 1993; Duluga and Masson, 2000; Oswald et al., 2015; Walsh et al., 2018). Thus, the investigation of birth cohort changes in college students' SWB has potential significance.

Compared with a conventional meta-analysis, a meta-analysis conducted in a cross-temporal manner does not calculate the effect size of every raw article but examines variations in the average points obtained in psychological measures over time (Twenge, 2011; Liu and Xin, 2015; Donnelly and Twenge, 2017). As SWB is frequently measured by the General Well-being Schedule (GWB) (Duan, 1996) in research conducted in China, in order to perform a cross-temporal meta-analysis, we collected data from original studies that reported GWB scores for Chinese college students. By recording the year of the study and participants' mean GWB scores for a series of studies conducted with comparable samples, we were able to examine birth cohort differences (e.g., Xin and Zhang, 2009; Twenge and Foster, 2010) in a cross-temporal meta-analysis. For example, several researchers (e.g., Fang et al., 2018; Yang and Sun, 2019) have measured Chinese college students' SWB using the GWB, and the year in which each study was conducted represents the birth cohort. In the scatter plot, the average point for each measure was used as the data point, among which, the X-coordinates represent the data collection year, and the Y-coordinates stand for the SWB score; thus, variations in the level of SWB can be analyzed (Xin and Zhang, 2009).

Another advantage of a cross-temporal meta-analysis is that it can study the impact of macro social indicators on social mentality. Under the view of the happiness-income paradox, this study first investigates birth cohort changes in Chinese college students' SWB from 2002 to 2017 and their relationship to the development of GDP per capita. Xin et al. (2010) suggested that the effects of large-scale social changes on an individual's development should be assessed in terms of three constructs: (1) economic conditions, (2) social connectedness, and (3) overall threat. Using the data from the World Values Survey, Xu and Xia (2014) found that the factors of economics, work, and family exhibited important impacts on participants' SWB. In fact, the factors of work and family are indicators of overall threat and social connectedness, respectively. Because college students have not yet taken part in work, the overall threat they experience may be indicated by the university enrollment rate and unemployment rate. Previous literature has shown that married people have higher levels of SWB (Easterlin, 1974; Gohm et al., 1998; Brewer, 2006; Chi, 2016). Although most college students are not married, there is evidence that their parents' marital status can affect college students' SWB (Zhang, 2011; Zhang et al., 2015). For example, college students who were from single-parent families had lower levels of SWB than those from dual-parent families (e.g., Fang et al., 2018). In summary, this study also examined the relationships between Chinese college students' SWB and GDP per capita, the divorce rate, the university enrollment rate, and the unemployment rate. We expected that the analysis can shed more light on understanding changes in college students' SWB.

This study also aimed to examine gender differences in Chinese college students' SWB. Although plenty of studies have revealed gender differences between the SWB of female and male college students (e.g., Perez, 2012; Li et al., 2015; Lu et al., 2016; Fang et al., 2018; Yang and Sun, 2019), the results of this research have been inconsistent. For example, Li et al. (2015) found that females had higher SWB levels than males, whereas Yang and Sun (2019) found that male college students had higher levels of SWB. Therefore, the current study used a general meta-analysis to examine gender differences more comprehensively. Moreover, a meta-analysis conducted in a cross-temporal manner was carried out to compare changing patterns in the SWB of different genders.

## Methods

### Literature Search

The GWB (Duan, 1996) is considered the SWB measurement adopted most frequently in China. The GWB refers to an 18-item questionnaire exhibiting six subscales, namely, health concerns (2 items, e.g., “*Are you bothered by illness, physical discomfort, pain or fear of illness?”*), energy (four items, e.g., “*How do you feel in general?”*), gratification and life interest (two items, e.g., “*Are you happy, satisfied or happy in your life?”*), frustration or pleasant moods (3 items, e.g., “*Do you feel depressed?”*), emotion and behavior control (three items, e.g., “*Do you have any reason to doubt that you have lost your sanity or control over your behavior, conversation, thinking or memory?”*), and relaxation and stress (four items, e.g., “*Do you worry about your neuroticism or insanity?”*). The rating of items 1–14 follows a six-point scale, while that of items 15–18 complies with a ten-point scale. The overall score is acquired through the sum of the scores of all items after reversing the scores of the negative items. In terms of the respective items, respondents rate their feelings over the past month; among the results, a high score indicates a higher well-being level. Existing psychometric studies have confirmed that the GWB is reliable and valid (Peng and Zheng, 2011; Yu et al., 2016). Accordingly, the present study harvested data from original studies on SWB scores of Chinese college students to conduct the present cross-temporal meta-analysis.

The literature search was conducted using three scholarly literature databases in Chinese, including Wangfang, China National Knowledge Internet, and Chongqing VIP Information. They have been mostly used in China and include Chinese journals of science and social science published after 1985 (such as psychology and associated subjects), as well as the master's papers and the doctoral dissertations published since 1995. The study terms employed for identifying the included studies were “subjective well-being,” “GWB,” and “college students.” In every article, the mean SWB score, publication year, and sample size were extracted to carry out the cross-temporal meta-analysis. In addition, the data collection year, which indicated the birth cohort of the participants, was also set as 2 years before the publication of the thesis if the specific time of data collection was not mentioned in the article (Xin and Zhang, 2009).

### Inclusive Rules

**Figure 1:**
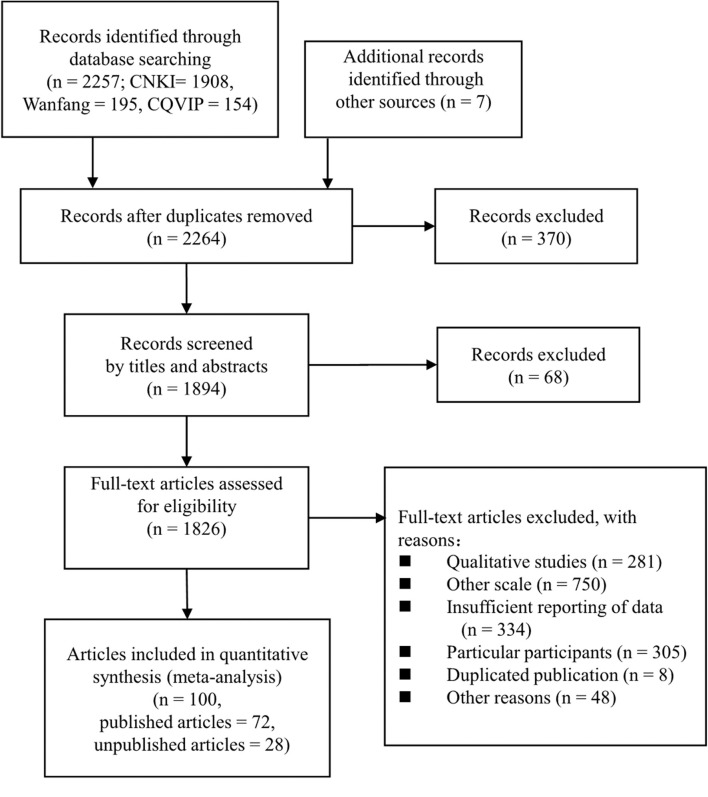
PRISMA flow chart for study selection.

For this meta-analysis, the study inclusion criteria were as follows:

(1) All of the involved participants were Chinese college students from routine 4-years faculties (e.g., students from 2-year colleges and military colleges or institutions were excluded). (2) All the participants were from mainland China. (3) The study involved no fewer than 30 males or females as the participants. (4) The mean value, standard deviation, and total sample size or unchosen subgroups were reported. (5) For identical datasets that were published twice, the original one was chosen. (6) No participant was screened based on certain standards (e.g., they were not selected to rate low or high score upon the other scale or were not clients at a counseling institute). The method, results, and the inclusion process have been detailed in the PRISMA diagram (Moher et al., 2009). As Figure 1 explains, we identified 2,257 articles via searches and removed 370 duplicates during the initial screening. We set aside 68 articles by screening titles and abstracts. The remaining 1,826 were screened in accordance with our inclusion criteria, and 100 articles were included in the analysis.

Finally, 100 studies (106 data points, *N* = 55,830) from 2002 to 2017 were obtained (Table 1). More information of the studies was depicted in the supplementary materials (Table S1). Among them, 57 studies reported statistical results for different genders and were used to examine possible gender differences. More details about those enrolled articles is provided in the Supplemental Material.

**Table 1:** Characteristics of the study samples from 2002 to 2017.

**Year of data collection**	**Number of articles**	**Sample size**	**Number of articles reporting gender differences**
2002	1	268	1
2004	2	1,102	0
2005	6	2,093	4
2006	2	585	0
2008	6	2,417	6
2009	5	1,418	4
2010	4	2,159	4
2011	12	5,116	6
2012	14	5,801	7
2013	13	10,375	6
2014	17	8,377	12
2015	6	3,692	3
2016	9	10,364	3
2017	3	2,036	1

### Control Variables

In previous cross-temporal meta-analyses (such as Xin and Zhang, 2009; Xin and Xin, 2016; Yang and Xin, 2019), regional differences and publication type were controlled for because these variables might be confounding factors of GWB data. According to region, data were divided as five types, including eastern, northeastern, middle, western, and mixed (studies that contained participants from two or more regions). Every article was divided into one of the following publication classes: (a) class one, which included the Science Citation Index (SCI), Social Science Citation Index (SSCI), and Chinese Science Citation Index (CSSCI); (b) class two, which included publications from other sources; and (c) class three, which included master's theses and dissertations.

### Sources for Corresponding Social Indicators

According to the findings of former studies (Xin and Xin, 2016; Yang and Xin, 2019), we chose the GDP per capita as one indicator of economic conditions, the divorce ratio as an indicator of social connectedness, and the preferred university enrollment rate and unemployment as two indicators of the overall threat. These data were identified from the China Statistical Yearbook (National Bureau of Statistics of China, 2017), except for the university enrollment rate, which was obtained from the national statistical bulletin of education development issued by the Ministry of Education (Bulletin of National Education Development Statistics, 2019).

### Data Analysis Methods

The current work is a cross-temporal meta-analysis. First, compared with a conventional meta-analysis, a meta-analysis performed with cross-temporal approach does not involve calculation of the study effect size but involves determination of the variation in the average psychological measure score over time (Twenge and Campbell, 2001). For the purpose of calculating the degree of the variation in SWB scores over time, the average specific year score (the initial and final years of those enrolled articles) was calculated using the following weighted regression equation: y = *B*x + *C* (where *B* indicates the sample size weighted based on the unadjusted regression coefficient, x indicates the year, and *C* indicates the sample size-weighted constant or intercept, whereas y indicates the estimated average SWB score).

Consistent with previous studies (Twenge et al., 2004; Twenge and Im, 2007; Xin and Zhang, 2009), the average standard deviation was computed by calculating the average of within-sample SDs mentioned in those data sources (representing the average variance within an individual sample). Notably, this method avoids ecological fallacy or alarming relevance (Twenge et al., 2004); these occur when the calculations of the magnitude of change are made based on the average score variation rather than the individual population variance. Such results aggravate the magnitude, since the average score shows less difference than the individual score. By contrast, that approach adopted in this study applied the SD from individual articles to capture the scale variance across individuals. Therefore, ecological falsehood was not a problem, since the effect size is based on the variance among individuals (Twenge et al., 2004; Twenge and Im, 2007).

Then, a time lag analysis was carried out in light of prior studies (Xin and Xin, 2016; Yang and Xin, 2019) in order to decide whether three patterns of social indicators were able to account for the changes in SWB. One indicator for economic conditions (GDP per capita), one indicator for social connectedness (divorce rate), and two indicators of overall threat (university enrollment rate, unemployment rate) per year were matched with the mean SWB for each study in that year in two ways: to the data collection year and to the three previous years of data collection. For instance, each study's data point for SWB scores that were collected since 2015 were matched with every social indicator from 2012 to 2015. Four regression analyses on each social indicator were implemented to weight the sample size. We reported the standardized βs, revealing the correlation between social indicators and SWB and weighted through the sample size. Based on the study by Twenge (2000), provided that social indicators are capable of explaining the changes in SWB over the past years, the connection should be of significance if SWB scores can be matched with social indicators from the previous several years. In addition, given that SWB and social indicators share corresponding relevance, notable correlations should be made between the two variables in the data collection year.

To investigate gender differences, based on ordinary meta-analysis, male students were regarded as the experimental group, while female students were regarded as the control group. The average effect size was calculated with the following formula:

$$\begin{array}{l} \bar{d}=\sum W_{i}d_{i}/\sum W_{i}\\ W_{i}=2N_{i}/(8+d_{i}^{2})\\ d=\frac{M_{female}-M_{male}}{SD}\\ \text{SD=}\sqrt{\left(n_{e}-1)S_{e}^{2}+(n_{c}-1)S_{c}^{2}/(n_{e}+n_{c}-2\right)} \end{array}$$

Where *W*_i_ indicates the study weight, *N*_i_ represents the overall sample size, *d* indicates the effect size of an individual article, SD represents the pooled SD in control and experimental groups, and Se/Sc and ne/nc indicate the sample size/SD in the control and experimental groups, respectively.

## Results

### Relationship Between Mean SWB Scores and Year

**Figure 2:**
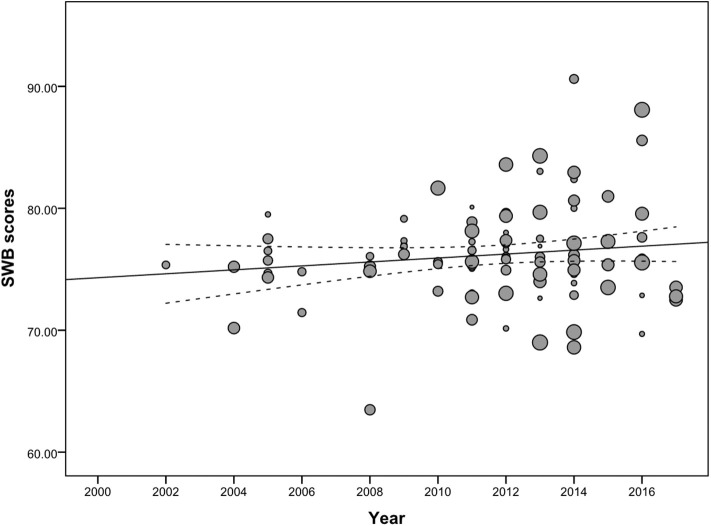
Changes in Chinese college students' SWB scores, 2002-2017.

How did the SWB of Chinese college students change from 2002 to 2017? Figure 2 shows a scatter plot regarding the observed mean SWB scores from 2002 to 2017, and the dot size is in proportion to those weights assigned by the above observed means for meta-regression. Additionally, the regression line and corresponding 95% confidence interval are shown. The plot shows an upward tendency in the SWB regression line; namely, from 2002 to 2017, there was an increase in the SWB of Chinese college students.

The data collection year was remarkably and positively related to SWB scores when weighting the sample size (β = 0.34, *t* = 2.18, *p* < 0.05). Similar results were obtained when weighting the sample size and controlling for the publication and regional variables (β = 0.40, *t* = 2.43, *p* < 0.05). However, there was no significant correlation of SWB scores with the data collection year squared (see Table 2). These findings show increasing trends of SWB scores among Chinese college students over the past year, revealing an impact exerted by birth cohort.

**Table 2:** Results of regression analyses of SWB.

**Predictors**	**SWB score with controls**			**SWB score without controls**		
		***SE***	**95% CI**		***SE***	**95% CI**
Year	0.46^*^	0.18	[0.11, 0.81]	0.41^*^	0.17	[0.07, 0.75]
Year squared	0.04	0.04	[−0.04, 0.11]	0.04	0.04	[−0.04, 0.11]
Region	0.07	0.44	[−0.81, 0.94]			
Publication class	0.88	0.69	[−0.50, 2.25]			

* *p < 0.05*.

A total of 40 articles reported subscale scores of GWB from 2002 to 2017 (e.g., health concerns, energy, gratification and life interest, frustration or pleasant moods, emotion and behavior control, relaxation and stress); therefore, we further investigated distinct change in patterns of six subscales. After weighting the sample size, frustration or pleasant moods was positively associated with the data collection year (β = 0.26, *t* = 2.45, *p* < 0.05), and energy was marginally associated with the data collection year (β = 0.25, *t* = 1.89, *p* = 0.066). After weighting the sample size, the data collection year had a negative association with health concerns (β = −0.41, *t* = −6.16, *p* < 0.001), gratification and life interest (β = −0.27, *t* =-2.16, *p* < 0.05), and emotion and behavior control (β = −0.22, *t* = −2.43, *p* < 0.05). No significant relationship between the year of data collection and relaxation and stress was observed (β = 0.04, *t* = 0.69, *p* = 0.49).

### Magnitude of Change in SWB

Since a birth cohort change in SWB was uncovered, the effect size was evaluated. When the sample size was evaluated, the mean SWB scores rose from 72.47 in 2002 to 77.60 in 2017. Thus, the score was elevated by 5.13 points, or 0.45 SDs (*SD* = 11.40), indicating a medium effect (Cohen, 1992). To enhance readability, the change in SD was transformed to percentile scores. If college students in China in 2002 achieved the mean scores at the 50th percentile of the distribution, those in 2017 would achieve those scores at the 67th percentile (assuming as a normal curve). Furthermore, the *d* value (0.45) was transformed to be the yearly variance interpretation of 4.82%.

### Gender Differences in SWB

To explore the gender differences in birth cohort changes in SWB, 57 articles that reported SWB scores and SDs for both genders were adopted for the cross-temporal meta-analysis (N_male_ = 12,933, N_female_ =16,303). During the evaluation of the sample size, neither the SWB scores of female students (β = 0.07, *t* = 0.42, *p* =0.68) nor those of male students (β = −0.07, *t* = −0.38, *p* = 0.71) was significantly correlated with the year. Similar results were obtained when weighting the sample size and controlling for the publication and regional variables. A general meta-analysis also found a nearly non-existent mean effect size of the gender differences in SWB scores (*d* = 0.07), according to the guidelines of Cohen (1992). Namely, the SWB scores of the two genders did not differ significantly.

### Correlations Between SWB and Social Indicators

To ascertain how indicators of the social environment are associated with the SWB of Chinese college students, SWB scores were matched with social indicators at two time points: 3 years prior to the year of data collection, and the year of data collection. Lagging the social indicators elucidated the associations between the SWB of college students and the social environment at their relatively young age. If the social environment impacted the well-being variables, the mentioned relationships would be noticeable when the scores were matched to social indicators that lagged 3 years (Twenge, 2000; Xin et al., 2010).

As Table 3 shows, Chinese college students' SWB was significantly correlated with GDP per capita, the divorce rate, and the university enrollment rate at both time points. However, SWB was not correlated with the unemployment rate. Namely, higher levels of GDP per capita, divorce rates, and university enrollment rates indicate higher levels of SWB perceived by Chinese college students.

**Table 3:** Correlations between social indicators and GWB, weighted by sample size.

**Indicators**	**Three years prior**		**Actual year**	
		**95%CI**		**95%CI**
Divorce rate	0.29^**^	[0.11,0.46]	0.22^*^	[0.03,0.39]
Unemployment rate	0.04	[−0.15,0.23]	−0.19	[−0.37,0.00]
GDP per capita	0.22^*^	[0.03,0.39]	0.19^*^	[−0.00,0.37]
University enrollment rate	0.19^*^	[−0.00,0.37]	0.20^*^	[0.01,0.38]

* *p < 0.05*;

#x0002A;*: *p < 0.01; n = 106*.

## Discussion

A cross-temporal meta-analysis was carried out in this work to investigate variations in the SWB among Chinese college students. The results implied that the SWB levels of Chinese college students increased by 5.13, or 0.45 SDs, from 2002 to 2017. The SWB levels of female and male college students did not differ significantly. In addition, indicators of the social environment, including the GDP per capita, the divorce rate, and the university enrollment rate, were positively correlated with the SWB of college students.

Economic growth and human well-being are two essential goals that both countries and individuals strive for; thus, these are important for the Chinese government. The Chinese government has always held and acted based on the belief that the essential goal of government is to make the lives of Chinese people better and happier. On the one hand, the Chinese government endeavors to increase the economic income and quality of life of the Chinese. During the past decades, China's annual GDP has grown at a surprising average speed of ~10%. Specifically, the GDP per capita of China increased from 1,148 dollars in 2002 to 8,759 dollars in 2017 (World Bank, 2019). The poverty rate in rural China decreased from 49.8 percent in 2000 to 3.1 percent in 2017 (National Bureau of Statistics of China, 2018). On the other hand, the Chinese government endeavors to increase the mental health and happiness of the Chinese. For example, the Chinese government released the national pilot work plan for the construction of social psychological service system in 2018, which aims to promote self-esteem, self-confidence, ration and peace, and positive social mentality of citizens. According to the work plan, by the end of 2021, the pilot areas should have been establishing the social psychological service system, integrating the mental health service into the social governance system, such as building platforms for social psychological services and integrating mental health services into the evaluation index system of healthy cities (China Disease Prevention Control Center, 2018). In June 2015, Chinese government expounded the concept of “targeted poverty alleviation.” Specially, according to the specific reasons of poverty, all social forces should be mobilized to participate in poverty alleviation through relocation, ecological protection, education, and minimum living insurance policies (CCTV news, 2019b). Therefore, we would expect that the SWB of the Chinese increased during the past decades. Using a cross-temporal meta-analysis, this study revealed that Chinese college students' SWB increased from 2002 to 2017. The results were consistent with those of Dong (2016), who found that the SWB of Chinese rural residents increased from 2001 to 2013. Using a data set that contains measurements of Chinese college students' well-being from 2002 to 2017, the present study provides new evidence of the increase in well-being among Chinese people during recent decades.

To examine the causes of the changes in SWB, this study also investigated the relationships between Chinese college students' SWB and indicators of the social environment. The results first supported a positive relationship between SWB and GDP per capita. In addition to the famous happiness-income paradox, Easterlin and Sawangfa (2009) also suggested that “a given absolute increase in GDP per capita has a greater impact on SWB in a low-income than a high-income country.” China is a typical developing country, and it was reasonable to hypothesize that the SWB in China increased as its GDP per capita increased. This hypothesis was supported. Although Chinese college students' SWB was positively correlated with GDP per capita, this study could not confirm a causal relationship between the increase in GDP per capita and the increase in SWB. Further study may investigate the relationship after control for related variables, such as the trust and satisfaction with governmental institutions, using longitudinal data.

This study also found that Chinese college students' SWB was positively correlated with the university enrollment rate and divorce rate. The university enrollment rate increased from 63% in 2002 to 81% in 2017. This expansion in university enrollment has given students more opportunities to receive higher education, which may have improved their SWB (Chen, 2012). Although divorce is viewed as a negative factor of well-being (Amato, 2010; Chi, 2016), this study, as well as Dong's (2016) study, revealed a positive relationship between the divorce rate and well-being. However, it should be noted that most college students are not married. The results may indicate that college students' SWB was affected by their parents' marital values and status. According to Dong's (2016) view, the positive relationship between the divorce rate and SWB indicates that divorce is gradually becoming an active choice for pursuing happiness. Taken together, college students' SWB may be improved by positive marital behaviors of their parents.

Moreover, many previous studies have revealed gender differences between the SWB of female and male college students (e.g., Li et al., 2015; Lu et al., 2016; Fang et al., 2018; Yang and Sun, 2019); however, neither the cross-temporal meta-analysis nor the general meta-analysis in this study confirmed such gender differences. Luhmann et al. (2012) proposed that although life events may affect males and females differently, individuals tend to adapt to their newly found situations and return to their original baseline levels of well-being. Hyde (2005) reviewed many meta-analyses of gender differences across a wide array of outcomes and characteristics and also concluded that men and women are more similar than they are different. These views may explain the present study results. However, only 57 studies were involved in the meta-analysis of gender differences, which may limit the power of the analysis.

In our view, the most important result of this study is that Chinese college students' SWB increased with the development of GDP per capita from 2002 to 2017. However, the relationship between economic growth and well-being should be investigated further. First, according to the view of the diminishing marginal utility of income, the relationship between economic growth and well-being is moderated by a country's current economic situation (Frey and Stutzer, 2002; Layard, 2005; Easterlin and Sawangfa, 2009). Namely, economic growth is more important for low-income countries than for high-income countries. Therefore, whether the economic growth in present-day China will improve well-being should be investigated. Second, SWB is a culturally relevant concept. The results of both the current study and Dong's (2016) study were based on data from Chinese studies; hence, whether the results can be generalized to other countries should be investigated further. Third, we used a cross-temporal meta-analysis to examine data from a number of independent studies of the role of GDP on SWB in order to determine overall trends in China. Some critical thinking and future directions, in particular the use of articles with participants with broader age range, should be addressed. Whether the results can be generalized to participants with a broader age range should be investigated further. Fourth, divorce rate may affect SWB in two ways. On the one hand, the divorce rate, as an indicator of social values and environment, may affect SWB in macrosystem level (Zhou and Fan, 1999; Bronfenbrenner and Morris, 2006); on the other hand, the marital status experienced by individuals or the marital status of their parents can affect SWB (Brewer, 2006; Fang et al., 2018). These two ways are relatively independent, and our research discussed here is about the former one. However, this study is unable to reveal the specific mechanism of the effect. We also admitted the influence of economic income on happiness has the same limitation. Fifth, in regard to methodology, the method of cross-temporal meta-analysis misestimating effects has been challenged by some researchers (e.g., Rudolph et al., 2019). Therefore, we recommend that future research should consider the method proposed by Rudolph et al. (2019) to verify the reliability of the results.

## Conclusion

This study conducted a meta-analysis of birth cohort changes in Chinese college students' subjective well-being from 2002 to 2017. First, the mean score of subjective well-being increased by 5.13, from 72.47 to 77.60, with 0.45 standard deviations (SD = 11.40). Second, their subjective well-being was significantly correlated with social indicators (e.g., GDP per capita, divorce rate, and university enrollment rate) for the corresponding year and 3 years prior to the collection of subjective well-being data. The results showed that social changes play an important role in predicting changes in well-being. We found that China's economic development in the past few years promotes the college students' happiness, which supports Easterlin's view that “a given absolute increase in GDP per capita has a greater impact on SWB in a low-income than a high-income country.”

## Data Availability Statement

The datasets generated for this study are available on request to the corresponding author.

## Author Contributions

QS conceptualized and designed the study, collected and organized the data, conducted the analyses, and drafted the initial manuscript. GL reviewed the included articles, critically reviewed and revised the manuscript. All authors read and approved the final manuscript.

### Conflict of Interest

The authors declare that the research was conducted in the absence of any commercial or financial relationships that could be construed as a potential conflict of interest.
