# Supplementary material for: Birth Cohort Changes in the Subjective Well-Being of Chinese College Students: A Cross-Temporal Meta-Analysis, 2002–2017
Source: Front Psychol. 2020 Jun 11;11:1011. doi: 10.3389/fpsyg.2020.01011 (PMC7300275; doi:10.3389/fpsyg.2020.01011)
Supplement: Supplementary file 1 [file Data_Sheet_1.docx]

Supplementary material

Literature that included in the meta-analysis:

^*^Ai, T. T., Ou, Y. M. K., & Lei, X. Z. (2011). Research on subjective sense of happiness and personality of college students. *Health Medicine Research and Practice, 8*(3), 42-46.

^*^An, G. H., Zhang, Y. L., & Shi, Y. S. (2013). A study on the relationship between social support and subjective well-being of college students. *Journal of Longdong University, 24*(1), 90-92.

^*^Chao, C. Y. (2013). The orientation of College Students' view of happiness and its relationship with subjective well-being and personal unhappiness. *Journal of Hubei University of Economics (Humanities and Social Sciences), 10*(4), 24-26.

^*^Chen, H. (2010). *A study on the negative emotion regulation strategies of college students and their correlation with subjective well-being* (Unpublished master’s thesis). Southwest University, Chongqing, China.

^*^Chen, H., Zeng, Y. Y., & Wu, J. Y. (2015). A survey of college students’ academic cheating and its relationship with subjective happiness. *Journal of Nanchang Institute of Technology, 34*(5), 62-65.

^*^Chen, X. H., Wang, X. G., & Lin, X. M. (2011). An investigation of sense of humor and subjective well-being and depression. *Health Medicine Research and Practice, 8*(1), 35-39.

^*^Cheng, Y. T. (2013). Study on the role of physical exercises in subjective well-being of university students. *Journal of Chongqing University of Education, 26*(6), 138-146.

^*^Cui, K. (2007). *The influence of family rearing style on achievement motivation and subjective well-being of college students* (Unpublished master’s thesis). Zhengzhou University, Zhengzhou, China.

^*^Cui, L. H. (2016). Study on the influence of social relationship satisfaction on subjective well-being of college students. *Chinese Incubator, 22*, 49-52.

^*^Deng, Z. J., Huang, H., Gui, Y. F., Niu, L. Y., & Zhou, C. Y. (2015). Mobile phone dependence, parenting style and subjective well-being in college students. *Chinese Mental Health Journal, 29*(1), 68-73.

^*^Fang, Z. W., Qin, Q., Chen, Y. Z., & Chen, J. (2013). Relationship analysis of college students’ self-esteem, interpersonal relationship & subjective well-being——Take chengdu as an example. *Journal of Chengdu University (Social Sciences), 2,* 123-126*.*

^*^Fang, J. C., Zheng, D., Zhi, X. L., & Xie, J. (2018). The distribution and correlation of general well-being and personality characteristics of undergraduates in Tianjin. *China Journal of Health Psychology, 26*(6), 916-920.

^*^Fang, Y. L. (2015). *Research on the relationship between personality characteristics, life events and subjective well-being of independent college student* (Unpublished master’s thesis). Central China Normal University, Wuhan, China.

^*^Gong, X. F. (2007). *The subjective well-being and its self-discrepancy correlation research of sports universities students* (Unpublished master’s thesis). Wuhan Institute of Physical Education, Wuhan, China.

^*^Guo, X. X. (2015). Relationship between Family Function, Self-esteem and Subjective Well-being among College Students. *China Journal of Health Psychology, 23*(4), 551-555.

^*^Guo, X. X. (2017). Study on the relationship between family function self-efficacy and subjective well-being of college students. *Journal of Campus Life & Mental Health, 15*(5), 328-330.

^*^Guo, Y. (2011). A study on the subjective well-being of Normal University Students. *China Electric Power Education, 1*, 141-143.

^*^Han, L., Liao, C. J., & Zhang, J. H. (2016). Stressful life events and subjective well-being: multiple mediating modes of emotion regulation and resilience. *Chinese Journal of Special Education (Monthly), 11*, 75-81.

^*^He, M. F. (2016). Investigation and analysis on subjective well-being of nursing students with only child and non-only undergraduate students. *Chinese General Practice Nursing, 14*(13), 1381-1382.

^*^He, T. F., Dong, C. Y., & Liu, W. Y. (2014). Experimental research of college students’ gratitude psychology on subjective well-being. *Occupation and Health, 30*(8), 1111-1116.

^*^Hou, J. B., Zhou, C. Y., Zhang, Y. Q., & Ye, T. (2018). Longitudinal study on mental health of college students. *Journal of Yangtze University (Social Sciences), 41*(3), 100-107.

^*^Hu, H. D., Zhang, Y., Gao, Y. T., & Zhu, X. (2018). Relationship of learning burnout and subjective well-being of military medical university students and the mediating role of psychological capital. *Academic Journal of Second Military Medical University, 39*(11), 1284-1287.

^*^Hu, Q. (2016). The study on the relationship between the university students’ emotion management ability and subjective well-being. China West Normal University.

^*^Huang, J. X., Wang, T, T., & Shi, Y. K. (2017). An analysis of the correlation between college students’ general happiness and suicidal ideation. *Journal of Suzhou University, 32*(5), 45-48.

^*^Huang, Y. (2014). *A research on college students’ subjective well-being and interpersonal trust* (Unpublished master’s thesis). Guangxi Normal University, Guilin, China.

^*^Huang, Y. Q. (2019). *Study on the relationship between college students' sense of belonging, school adaptation and general well-being* (Unpublished master’s thesis). Guangxi Normal University, Guilin, China.

^*^Jiang, L. L., Feng, J. Q., & Li, Y. Y. (2010). An investigation on subjective well-being of undergraduates in Hangzhou. *Health Research, 30*(6), 442-445.

^*^Jiang, N. (2013). *Research on the relationship between college students’ dormitory interpersonal relationship and self-consistency and congruence and subjective well-being* (Unpublished master’s degree). Inner Mongolia Normal University, Huerhaote, China.

^*^Jiang, T., & Liu, H. (2016). Influence on subjective well-being of college students imposed by personality and coping styles. *Journal of Mudanjiang Normal University, 5*, 120-124.

^*^Jing, L. T. (2017). *The research on the correlation between subjective well-being of college students and materialistic values* (Unpublished master’s thesis). Guangxi Normal University, Guilin, China.

^*^Kong, D. S., & Zhang, W. (2007). Relationship between life events, way of coping, social support and subjective well-being of impoverished college students. *Chinese Journal of Clinical Psychology, 15*(1), 61-63.

^*^Li, C. (2012). *The analysis on the relationship among the employment stress, the resilience of job select and subjective well-being of college students* (Unpublished master’s degree). Harbin Normal University, Harbin, China.

^*^Li, F. H., Liu, Q., Long, L. L., Zhao, Y., & Feng, S. D. (2016). The relationship among coping style, self- efficacy and subjective well-being of university students. *China Journal of Health Psychology, 24*(9), 1320-1324.

^*^Li, M. M., Zheng, X. F., Zheng, D., & Guo, D. X. (2015). Multidimensional analysis of college students’ happiness in Ningbo. *Journal of Ningbo Institute of Education, 17*(2), 33-37.

^*^Li, Q. (2011). Research on college students' sense of happiness based on the theory of social reaction. *Journal of Shanxi College for Youth Administrators, 24*(1), 29-31.

^*^Li, R. L., & Chen, Y. H. (2006). Research on the relationship between comprehension of social support and subjective well -being of medical students. *Journal of North Sichuan Medical college, 21*(4), 371-373.

^*^Li, S. (2014). Group counseling on improving the effect of College Students’ academic procrastination. *Science of Social Psychology, 29*, 10-14.

^*^Li, W. H., Wang, L. P., & Yuan, J. (2014). Correlation of family cohesion, family adaptability and subjective well-being in college students. *China Journal of Health Psychology, 22*(7), 1067-1069.

^*^Li, Y. (2007). The relativity research of feeling of happiness of college student and their physical endowment health in the school. *Journal of Nanjing Institute of Physical Education, 21*(6), 32-34.

^*^Li, Y. (2012). *The study of the relationship between nostalgia and the subjective well-being of university students* (Unpublished master’s degree). Southwest University, Chongqing, China.

^*^Lin, J. G. (2007). *Investigation on relationship between Fujian college students’ subjective well-being and upbringing style of their parents* (Unpublished master’s thesis). Guizhou Normal University, Guiyang, China.

^*^Lin, M. M., Liu, X. Y., Long, C. X. (2018). A study on the relationship between forgiveness level and subjective well-being of college students. *Course Education Research, 9*, 189-190.

^*^Liu, D. Y. (2017). *An empirical study on the influence of College Students’ gratitude tendency on subjective well-being* (Unpublished master’s thesis). Shanxi Normal University, Linfen, China.

^*^Liu, S. (2015). Research on the relationship between parental rearing patterns, mental resilience and subjective well-being in engineering college (Unpublished master’ thesis). Tianjin University, Tianjin, China.

^*^Lu, Z. F., Han, M., Ma, J. M., & Wang, P. P. (2016). The relationship between college students’ goals pursuit and their general well-being. *Psychology: Techniques and Applications, 4*(2), 71-77.

^*^Ma, X. W. (2008). *The research about Tibetan students emotional intelligence, self-efficacy and subjective well-being* (Unpublished master’s thesis). Northwest University, Xian, China.

^*^Mai, M. T., & Yao, B. X. (2016). A study on the self-happiness of college students. *Educator, 7*, 78-80.

^*^Mei, Y. Y., Pang, S. Q., Zheng, L. X., Li, W. T., Xin, H. M., & Liu, L. Y. (2016). Correlative study on competency, self-efficacy and general well-being among undergraduate nursing students. *International Journal of Nursing Practice, 35*(13), 1841-1845.

^*^Nian, J., Jin, Y. B., Ren, Y. L., & Tao, L. Z. (2015). Achievement motivation and subjective well-being among college students: the mediation role of time management disposition. *China Journal of Health Psychology, 23*(2), 249-252.

^*^Nie, J., Jin, Y. B., Ren, Y. L., & Tao, L. Z. (2015). Achievement motivation and subjective well-being among college students: the mediation role of time management disposition. *China Journal of Health Psychology, 23*(2), 249-252.

^*^Nie, Y. Q. (2014). Research on the relationship between college students’ resilience， coping style, social support and subjective well-being. Hunan Normal University.

^*^Niu, Y. (2018). Investigation on the subjective well-being of college students. *Folder*, *23*, 168-169.

^*^Peng, J., & Li, X. Y. (2014). A study on the relationship between college students’ happiness and psychological capital. *Journal of Inner Mongolia Normal University (Educational Science), 27*(10), 109-110.

^*^Peng, X. H., & Zheng, Y. Z. (2011). The relationship between College students’ subjective well-being and coping style. *Science of Social Psychology, 26*, 240-246.

^*^Qian, F. (2014). Study on the relationship between personality traits，emotional awareness capability and SWB of college student. *Science of Social Psychology, 29*, 5-9.

^*^Qu, F. (2018). *A study on the intervention of Thanksgiving group activities to improve the subjective well-being of College Students* (Unpublished master’s thesis). Hebei Normal University, Shijiazhuang, China.

^*^Ren, L. B., & Wang, H. X. (2015). A study on the relationship of college students’ psychological resilience, negative emotion and happiness. *Journal of Jimei University, 16*(1), 36-40.

^*^Rong, T. T. (2012). Relational research of university students' life emotional intelligence and self-consistency and congruence and subjective well-being (Unpublished master’s thesis). Jinan University, Guangzhou, China.

^*^Ruan, B. H. (2017). Investigation and analysis on subjective well-being and influencing factors of female college students’ left-behind experiences. *Journal of Educational Development*, 35-39.

^*^Shan, C., Wang, Y., & Liu, X. H. (2010). Research on the relationship between learning burnout and subjective well-being of college students. *China Journal of Health Psychology, 18*(8), 951-954.

^*^Shang, H. T. (2014). A study on the relationship between Internet addiction and subjective well-being of college students. *Journal of Sanmenxia Polytechnic, 12*(3), 31-35.

^*^Song, S. J. (2018). Survey on general self-efficacy and subjective well-being of science and engineering college students-A case study of Zhejiang University of Science and Technology. *Journal of Zhejiang University of Science and Technology, 30*(2), 143-148.

^*^Sun, Y., & Ji, W. (2018). The relationship between attribution style, innovative self-efficacy and subjective well-being of college students. *Chinese Rural Health Service Administration, 38*(7), 952-953.

^*^Tian, L., Wang, X. Q., & Hu, F. X. (2007). Development characteristics of normal university students’ subjective well-being. *China Journal of Health Psychology, 15*(6), 523-525.

^*^Wan, Z. W., Qin, Q., Chen, Y. Z., & Chen, J. (2013). Relationship analysis of college students’ self-esteem, interpersonal relationships & subjective well-being. *Journal of Chengdu University, 2*, 123-126.

^*^Wang, F. (2013). *The research of relationship among values, interpersonal relationship and subjective well-being of college students* (Unpublished master’s thesis). University of Jinan, Jinan, China.

^*^Wang, H., & Liu, H. (2016). The Relationship between Self-concept and Subjective Well-being of College Students. *Journal of Hubei Correspondence University, 29*(11), 47-48.

^*^Wang, L. (2018). On the relationship between academic pressure and self-contentment of college students: the role of resilience as a mediator. Hunan Normal University.

^*^Wang, X. M. (2013). *Relationship research on college students’ coping styles time management disposition, self-harmony and the subjective well-being* (Unpublished master’s thesis). Henan Normal University, Xinxiang, China.

^*^Wang, Y. M. (2013). *The related relationship and intervention study of psychological health of college students and explanatory style and subjective well-being* (Unpublished master’s thesis). Liaoning University, Shenyang, China.

^*^Wang, Y. N. (2004). *The relations between subjective well-being and locus of control among college students* (Unpublished master’s thesis). Jilin University, Changchun, China.

^*^Wang, Y., & Wang, Z. H. (2013). Resilience of college students and the relations of resilience to positive emotion and to subjective well-being. *Psychological Development and Education, 1*, 94-100.

^*^Wu, J., Xu, M. D., Lin, S. L., & Zhang, X. N. (2010). Study on the relationship between self-efficacy and general well-being of college students in Guangzhou higher education mega center. *China Journal of Health Psychology, 18*(8), 955-958.

^*^Wu, Y. G., Wei, X. N., Li, G. M., Zhang, C., & Zheng, L. (2016). The relationship between college students’ EQ and their SWB and self-esteem in Guangxi. *China Journal of Health Psychology, 24*(2), 244-247.

^*^Xiong, S. P. (2014). The effect of new mode of positive mental health education for College Students on learning burnout and subjective well-being. *Emotional reader*, *2*, 33-36.

^*^Xiao, W., & Xiao, Q. (2006). Factors impacting on social adaptability of undergraduates in city of Xi’an. *Journal of the Fourth Military Medical University*, *27*(4), 344-346.

^*^Xu, C. M. (2013). *Experimental study of promoting sports professional college students’ happiness* (Unpublished master’s thesis). Zhengzhou University, Zhengzhou, China.

^*^Xu, H. (2010). *A study of the relationship of time management disposition, subjective well-being, psychological stress and mental health* (Unpublished master’s thesis). Central South University, Changsha, China.

^*^Xu, L. L., & He, W. (2016). The relationship between curiosity and happiness of college students: the intermediary role of gratitude. *Science of Social Psychology, 31*(2), 39-43.

^*^Xu, S. H. (2013). *The study on relationship between subjective well-being and general self-efficacy of college students* (Unpublished master’s thesis). Qinghai Normal University, Xining, China.

^*^Yang, D. H. (2008). A study on the relationship between the subjective well-being of normal university students and the style of study in the dormitory. *Journal of Hubei Normal University, 28*(1), 113-115.

^*^Yang, S. Q., & Zhang, J. X. (2012). Relationship between dormitory interpersonal relationship and subjective well-being of college students. *China Journal of Health Psychology, 20*(12), 1905-1907.

^*^Yang, X. M., Qi, Y. L., Shen, Z. F., Han, B. X., & Meng, B. (2015). Family socioeconomic status, vocational value on subjective well-being in medical students. *Chinese Journal of Clinical Psychology, 23*(1), 154-158.

^*^Yang, X. M., Qi, Y. L., Shen, Z. F., Han, B. X., & Meng, B. (2015). Relationships among personality characteristics, interpersonal trust, and subjective well-being of medical students. *Journal of Shanghai Jiao Tong University Medical Science, 35*(1), 102-106.

^*^Yang, Y., & Sun, S. X. (2019). A study on the subjective well-being and mental health of college students with left-behind experiences. *Journal of Xichang University (Natural Science Edition), 33*(1), 92-95.

^*^Yao, Q. (2015). *A study on the relationship among subjective well-being, coping style and psychological flexibility of college volunteers* (Unpublished master’s thesis). Yangzhou University, Yangzhou, China.

^*^Ye, F. F., Xie, M. F., Wang, L. J. Wang, S. L., & Li, L. (2018). Studying on the relationship between work hoped-for selves and subjective well-being mediated by resilience of college students. *Chinese Health Service Management, 12*, 938-941.

^*^Ye, Y. H. (2014). A study on the relationship of social support and subjective well-being of college students: mediation effect of coping style. *Journal of Huzhou University, 36*, 83-88.

^*^Ye, Y. H., & Liu, Y. C. (2014). Relationship of fear of negative evaluation，interpersonal disturbances and subjective well-being in college students. *Journal of Chongqing University of Technology (Social Science), 28*(7), 140-145.

^*^Yu, X. D., Liu, W. X., & Li, Y. M. (2016). The influences of volunteering tendency and perceived social support on well-being among college students. *Journal of Adult Education of Hebei University, 18*(2), 109-113.

^*^Zhan, D. D. (2016). The influencing factors of subjective well-being of college students and the improvement measures. *Reading Abstract*, *11*, 128-129.

^*^Zhang, H. L., Su, J. P., & Li, Q. (2016). The relationship between subjective well-being and personality characteristics of college students. *China Market, 31*, 265-266.

^*^Zhang, H. L., Su, J. P., Li, Q., & Ma, Z. (2015). The relationship between college students subjective well-being and mental health. *Journal of Mudanjiang Normal University, 4*, 110-112.

^*^Zhang, H., Zhou, Y., & Dai, B. (2014). Researches on the medical college students’ gratitude, subjective well-being and the correlativity between them in Chengdu. *Journal of Chengdu Medical College, 9*(5), 624-627.

^*^Zhang, J. Y. (2013). The research on college students’ school adaptability and subjective well-being. *Journal of Guizhou Normal University (Natural Sciences), 31*(1), 37-40.

^*^Zhang, T., Jiang, R., & Gao, C. (2016). Subjective well-being of TCM college students and its relationship with life events. *China Journal of Health Psychology, 24*(12), 1812-1813.

^*^Zhang, X. Z. (2014). Private college students’ subjective well-being. *China Journal of Health Psychology, 22*(9), 1414-1418.

^*^Zhao, Y. R., & Jiang, S. Z. (2015). A study on the relationship between self-efficacy and subjective well-being of college students. *Journal of Education Pilot, 9*, 11-13.

^*^Zhou, C. Y., Huang, H., Liu, C. L., & Wu, H. M. (2014). Impact of left-behind experience in childhood on undergraduates’ subject well-being: the moderating role of parent emotional warmth. *Chinese Journal of Clinical Psychology, 22*(5), 893-896.

^*^Zhu, H., Yin, K. L., Song, Y. C., & Liang, Y. L. (2019). The relationship between college students’ boredom, subjective well-being, cognitive failure experience and online game addiction. *Journal of Dali University, 4*(3), 110-115.
