# Supplementary material for: Birth Cohort Changes in the Subjective Well-Being of Chinese College Students: A Cross-Temporal Meta-Analysis, 2002–2017
Source: Front Psychol. 2020 Jun 11;11:1011. doi: 10.3389/fpsyg.2020.01011 (PMC7300275; doi:10.3389/fpsyg.2020.01011)
Supplement: Supplementary file 2 [file Data_Sheet_2.docx]

Table S1. Description of subjective well-being studies included in the overall meta-analysis

| Authors | Publication year | N | Publication class | Region | M | SD |
| --- | --- | --- | --- | --- | --- | --- |
| Wang | 2002 | 268 | 3 | 2 | 75.36 | 13.17 |
| Xiao & Xiao | 2004 | 549 | 2 | 4 | 75.21 | 12.95 |
| Li & Chen | 2004 | 553 | 2 | 4 | 70.18 | 7.56 |
| Cui | 2005 | 373 | 3 | 3 | 75.72 | 12 |
| Kong & Zhang | 2005 | 163 | 1 | 2 | 79.5 | 10.6 |
| Gong | 2005 | 446 | 3 | 5 | 77.5 | 6.47 |
| Lin | 2005 | 550 | 3 | 1 | 74.33 | 6.12 |
| Tian et al. | 2005 | 289 | 1 | 3 | 74.68 | 10.72 |
| Ma | 2006 | 292 | 3 | 4 | 71.45 | 9.68 |
| Chen | 2008 | 634 | 3 | 3 | 74.84 | 10.18 |
| Shan et al. | 2008 | 267 | 1 | 3 | 76.07 | 11.18 |
| Jiang et al. | 2008 | 491 | 2 | 1 | 75.23 | 10.57 |
| Wu et al. | 2008 | 461 | 1 | 1 | 63.48 | 0.4 |
| Peng & Zheng | 2009 | 246 | 2 | 1 | 76.87 | 12.01 |
| Ai et al. | 2009 | 247 | 2 | 3 | 79.14 | 8.96 |
| Wang & Wang | 2011 | 207 | 1 | 3 | 78.49 | 13.07 |
| Wan et al. | 2011 | 862 | 2 | 4 | 75.63 | 11.43 |
| An et al. | 2011 | 246 | 2 | 4 | 73.04 | 12.09 |
| Chen | 2011 | 293 | 2 | 4 | 76.55 | 11.92 |
| Xu | 2011 | 233 | 3 | 4 | 77.26 | 10.57 |
|  |  |  |  |  |  | (continued) |
| Table S1 Continued | | | | | | |
| Authors | Publication year | N | Publication class | Region | M | SD |
| Zhang et al. | 2012 | 370 | 2 | 4 | 75.82 | 12.54 |
| Ye & Liu | 2012 | 569 | 2 | 4 | 77.37 | 10.88 |
| He et al. | 2012 | 178 | 2 | 3 | 76.63 | 10.61 |
| Peng & Li | 2012 | 277 | 2 | 4 | 77.09 | 5.89 |
| Li et al. | 2012 | 100 | 1 | 3 | 78.01 | 10.28 |
| Qian | 2012 | 277 | 2 | 1 | 76.06 | 9.96 |
| Zhou et al. | 2012 | 900 | 1 | 3 | 83.59 | 11.13 |
| Zhang | 2012 | 385 | 1 | 3 | 79.61 | 11.89 |
| Li | 2012 | 240 | 2 | 2 | 79.37 | 11.6 |
| Shang | 2012 | 168 | 2 | 4 | 70.15 | 6.96 |
| Nie et al. | 2013 | 64 | 1 | 1 | 74.56 | 6.29 |
| Nie et al. | 2013 | 88 | 1 | 1 | 73.82 | 8.21 |
| Nie et al. | 2013 | 70 | 1 | 1 | 72.63 | 7.33 |
| Nie et al. | 2013 | 86 | 1 | 1 | 74.57 | 5.82 |
| Guo | 2013 | 261 | 1 | 1 | 77.51 | 13.17 |
| Ren & Wang | 2013 | 55 | 2 | 4 | 80.03 | 10.91 |
| Ren & Wang | 2013 | 31 | 2 | 4 | 76.9 | 8.74 |
| Ren & Wang | 2013 | 42 | 2 | 4 | 77.47 | 9.09 |
| Ren & Wang | 2013 | 55 | 2 | 4 | 73.98 | 8.85 |
| Chen et al. | 2013 | 913 | 2 | 3 | 74.59 | 14.24 |
|  |  |  |  |  |  | (continued) |
| Table S1 Continued | | | | | | |
| Authors | Publication year | N | Publication class | Region | M | SD |
| Zhao & Jiang | 2013 | 190 | 2 | 3 | 79.91 | 11.81 |
| Fang | 2013 | 453 | 3 | 3 | 75.6 | 10.07 |
| Yang et al. | 2013 | 4054 | 1 | 3 | 69 | 13.95 |
| Li et al. | 2013 | 593 | 2 | 1 | 73.99 | 8.82 |
| Yang et al. | 2014 | 1519 | 2 | 3 | 69.85 | 13.45 |
| Deng et al. | 2013 | 1477 | 1 | 3 | 84.3 | 11.5 |
| Yao | 2013 | 994 | 3 | 1 | 79.68 | 10.85 |
| Liu | 2013 | 416 | 3 | 1 | 76.01 | 10.03 |
| Yu et al. | 2014 | 475 | 2 | 1 | 76.17 | 10.9 |
| Zhang et al. | 2014 | 605 | 2 | 2 | 82.95 | 10.57 |
| He | 2014 | 117 | 2 | 1 | 73.02 | 11.78 |
| Wu et al. | 2014 | 364 | 1 | 4 | 90.61 | 9.37 |
| Yang et al. | 2014 | 178 | 2 | 1 | 73.87 | 9.04 |
| Cui | 2014 | 580 | 2 | 3 | 75.72 | 11.45 |
| Han et al. | 2014 | 879 | 2 | 4 | 68.6 | 8.26 |
| Lu et al. | 2014 | 250 | 2 | 2 | 74.61 | 9.69 |
| Hu | 2014 | 1047 | 3 | 5 | 77.14 | 9.7 |
| Jiang & Liu | 2014 | 225 | 2 | 2 | 80 | 15.32 |
| Li et al. | 2014 | 487 | 1 | 3 | 80.64 | 11.98 |
| Zhan | 2014 | 244 | 2 | 3 | 82.38 | 12.68 |
|  |  |  |  |  |  | (continued) |
| Table S1 Continued | | | | | | |
| Authors | Publication year | N | Publication class | Region | M | SD |
| Wang & Liu | 2014 | 262 | 2 | 4 | 75.42 | 10.78 |
| Zhang et al. | 2014 | 310 | 1 | 3 | 72.88 | 11.34 |
| Liu | 2015 | 585 | 3 | 3 | 75.37 | 11.68 |
| Guo | 2015 | 256 | 2 | 1 | 77.55 | 13.22 |
| Huang et al. | 2015 | 950 | 2 | 3 | 77.28 | 11.85 |
| Ruan | 2015 | 565 | 2 | 4 | 80.98 | 10.51 |
| Jing | 2015 | 1158 | 3 | 4 | 73.51 | 11.12 |
| Sun & Ji | 2015 | 178 | 1 | 2 | 77.36 | 13.24 |
| Lin et al. | 2016 | 332 | 1 | 3 | 75.9 | 11.56 |
| Hou et al. | 2016 | 3701 | 2 | 3 | 88.08 | 13.59 |
| Ye et al. | 2016 | 418 | 1 | 1 | 77.62 | 11.11 |
| Niu | 2016 | 87 | 2 | 3 | 72.86 | 6.17 |
| Qu | 2016 | 462 | 3 | 3 | 85.57 | 7.65 |
| Hu et al. | 2016 | 745 | 2 | 4 | 79.56 | 13.86 |
| Song | 2016 | 129 | 2 | 1 | 69.7 | 1.87 |
| Fang et al. | 2016 | 4012 | 1 | 1 | 75.56 | 10.59 |
| Yang & Sun | 2017 | 748 | 2 | 3 | 72.78 | 10.23 |
| Zhu et al. | 2017 | 615 | 2 | 4 | 73.52 | 8.02 |
| Nie at al. | 2013 | 308 | 1 | 1 | 73.91 | 7.01 |
| Li | 2010 | 355 | 3 | 4 | 75.6 | 10.8 |
|  |  |  |  |  |  | (continued) |
| Table S1 Continued | | | | | | |
| Authors | Publication year | N | Publication class | Region | M | SD |
| Chen | 2008 | 264 | 3 | 3 | 75.09 | 10.31 |
| Chen & He | 2014 | 589 | 1 | 5 | 74.94 | 11.46 |
| Li | 2010 | 1006 | 3 | 2 | 81.66 | 13.83 |
| Rong | 2010 | 443 | 3 | 1 | 73.2 | 9.11 |
| Ye | 2012 | 432 | 2 | 1 | 74.93 | 12.33 |
| Wang | 2011 | 189 | 3 | 2 | 75.06 | 11.88 |
| Chao | 2011 | 843 | 2 | 3 | 72.71 | 14.47 |
| Yang & Zhang | 2010 | 355 | 1 | 1 | 75.42 | 12.28 |
| Zhang | 2011 | 464 | 2 | 4 | 70.86 | 6.67 |
| Wang | 2016 | 478 | 3 | 3 | 75.5 | 11.21 |
| A & Yang | 2014 | 246 | 2 | NA | 69.91 | 11.03 |
| Li | 2009 | 475 | 2 | 1 | 76.23 | 5.85 |
| Guo | 2009 | 228 | 2 | 4 | 77.34 | 12.48 |
| Chen et al. | 2009 | 222 | 2 | 3 | 77.01 | 11.59 |
| Yang | 2006 | 293 | 2 | NA | 74.8 | 9.11 |
| Xu | 2008 | 300 | 3 | 3 | 75.17 | 7.53 |
| Li | 2005 | 272 | 2 | 1 | 76.51 | 9.98 |
| Nie | 2012 | 721 | 3 | 3 | 79.37 | 10.09 |
| Jiang | 2011 | 368 | 3 | 4 | 75.32 | 8.73 |
| Huang | 2017 | 673 | 3 | 1 | 72.5 | 13.67 |
|  |  |  |  |  |  | (continued) |
| Table S1 Continued | | | | | | |
| Authors | Publication year | N | Publication class | Region | M | SD |
| Wang | 2011 | 928 | 3 | 3 | 78.14 | 11.4 |
| Huang | 2012 | 1034 | 3 | 4 | 73.03 | 10.46 |
| Zhang et al. | 2013 | 225 | 2 | 2 | 83.03 | 10.53 |
| Xu | 2011 | 34 | 3 | 3 | 80.1 | 6.46 |
| Wang | 2011 | 449 | 3 | 1 | 78.89 | 12.47 |
| Xiong | 2012 | 150 | 2 | 3 | 77.32 | 12.27 |

Note: N, sample size; Region: 1 = east; 2 = northeast; 3 = center; 4 = west; 5 = multiple; Publication class: 1 = core journal; 2 = publication from other academic sources; 3 = dissertations and master’s theses; NA, missing values; M, mean score of subjective well-being; SD, standard deviation of subjective well-being.
